# Exogenous sucrose alleviates salt stress in sunflower (*Helianthus annuus* L.) and canola (*Brassica napus* L.) by modulating osmotic adjustment and antioxidant defense system

**DOI:** 10.1007/s12298-025-01571-9

**Published:** 2025-03-19

**Authors:** Büşra Sevgi, Sema Leblebici

**Affiliations:** 1 Institute of Graduate Education, Department of Molecular Biology and Genetics, Bilecik Şeyh Edebali University, Bilecik, 11230 Türkiye; 2 Faculty of Science, Department of Molecular Biology and Genetics, Bilecik Şeyh Edebali University, Bilecik, 11230 Türkiye

**Keywords:** Antioxidant enzyme, Canola, Gene expression, Salt stress, Sucrose, Sunflower

## Abstract

Salinity, a major ecological problem worldwide, adversely affects plant growth and productivity. Osmoprotectants are a possible strategy for plants to cope with and regulate their response to unfavorable environmental conditions, such as salinity. However, the role of sucrose in this process requires more precise elucidation. This study aims to investigate the ameliorative role of sucrose on growth parameters, proline content, antioxidant enzyme activity, and gene expression in sunflower and canola under salt stress. The treatments included a 3% sucrose concentration and two levels of salinity (75 and 150 mM NaCl). Salinity caused a remarkable reduction in stem-root growth, chlorophyll amounts and catalase (CAT) activity, whereas it unchanged ascorbate peroxidase (APX) activity. Furthermore, both plants grown under salt stress had considerably higher total protein, proline, malondialdehyde (MDA) content, and superoxide dismutase (SOD) activity. Exogenous sucrose increased plant growth, chlorophyll amounts and the activities of hydrogen peroxide-detoxifying antioxidant enzymes such as CAT and APX in salt-stressed plants, but dramatically depressed levels of osmoregulators such as protein and proline. Besides that, it balanced antioxidant enzyme levels by regulating SOD activity to the required level, thereby facilitating the effective operation of the antioxidant defense system. Additionally, sucrose had a different effect on gene expressions of antioxidants in sunflower and canola under salinity. These results revealed that sucrose can ameliorate the deleterious effects of salinity in sunflower and canola by modulating osmotic substance accumulation, the activity of antioxidant enzymes, and their gene expression. In conclusion, sucrose can be a potential tool for plants in salt stress alleviation.

**Supplementary Information:**

The online version contains supplementary material available at 10.1007/s12298-025-01571-9.

## Introduction

Soil salinity, which occurs due to geological and anthropogenic reasons, is one of the most serious environmental stressors limiting the growth and yield of plants worldwide, especially in arid and semi-arid regions (Hernández 2019). Salinity not only creates osmotic stress in plants by preventing the uptake of water from the soil but also creates ionic stress through the toxic accumulation of sodium and chloride ions in the leaves. This adversely affects the germination and development of plants and may even result in the death of the whole plant (Parihar et al. 2015). Salt ions taken up from the soil by plant roots cause disruption of the osmotic balance in the cytosol. Under stress conditions, some osmotic adjustments are needed to restore the disturbed cell homeostasis, requiring the synthesis of osmotic protective compounds in plants (Isayenkov 2012; Pirasteh‐Anosheh et al. 2016). Amino acids such as proline, quaternary ammonium compounds, sugars, sugar alcohols, and polyamines are among the main compatible solutes that accumulate during stress (Zulfiqar et al. 2020). The biosynthesis and accumulation of these compatible compounds, including proline and soluble sugars, help protect plant cells from the deleterious effects of salinity (Munns and Tester 2008).

Soluble sugars, including sucrose, glucose, and trehalose, are involved in basic physiological processes in plants, such as respiration, photosynthesis, germination, flowering, and senescence. In addition to being essential energy sources and structural components for plant growth and metabolism, sugars also function as storage/transport and signaling molecules and play an effective role in the maintenance of cellular homeostasis and defense mechanisms in plants during stress (Ahmad et al. 2020). Exogenous application of sugars at low concentrations also promotes seed germination and flowering, regulates photosynthesis, and delays senescence in plants under unfavorable conditions (Sami et al. 2016). Therefore, the exogenous application of sugars, such as sucrose, glucose and trehalose, is mostly associated with enhanced tolerance of plants to abiotic stresses, especially salinity (Khan et al. 2020).

Sucrose (Suc), one of the primary products of photosynthesis, constitutes the most abundant solute in plants. It plays a central role in plant structure and metabolism, as well as being associated with plant stress resistance (Wind et al. 2010; Lunn 2016). It functions as a prominent signaling molecule involved in the regulation of several metabolic and developmental processes, including shoot branching and floral induction. As an important osmotic regulator, it can maintain the osmotic balance of plant cells and help stabilize their proteins and membranes (Lunn 2016). Moreover, it can eliminate reactive oxygen species (ROS) by directly quenching free radicals. Therefore, the modulation of sucrose concentration in plants can improve their tolerance to abiotic stresses such as salinity and drought (Gangola and Ramadoss 2018). A limited number of studies have reported that sucrose treatment can enhance the tolerance of plants to salinity (Siringam et al. 2012; Qiu et al. 2014; Wang et al. 2019).

Sunflower (*Helianthus annuus* L.), a member of the Asteraceae family, is a major fourth oilseed crop grown in more than 70 countries worldwide in temperate climates (Rauf et al. 2017). Canola (*Brassica napus* L.), which belongs to the genus *Brassica* from the Brassicaceae family, also ranks third among oilseed crops after palm and soybean (Goyal et al. 2021). Both plants are the most important oil-yielding crops that can be grown in many regions of the world for various purposes, such as edible vegetable oil, industrial oil, and animal feed. The deleterious impacts of salt stress on plants have been widely investigated and elucidated, and recent studies have focused on how to ameliorate salt-induced damages. Although exogenous osmoprotectant treatment is a widespread and leading strategy in this process, the studies examining the effect of sucrose, an osmoregulator disaccharide sugar, under salt stress are very limited. Hence, it was hypothesized that sucrose can alleviate the negative effects of salt stress on sunflower and canola. Keeping in view the importance of sunflower and canola, this study aims to examine the effects of 3% exogenous sucrose treatment under different concentrations of salinity (75 and 150 mM NaCl) on phenological growth parameters, proline content, antioxidant enzymes, and gene expression levels in both oilseed crops.

## Materials and methods

### Plant material and treatments

Suzuka seeds, the most widely grown sunflower (*Helianthus annuus* L.) variety in Turkey, and canola (*Brassica napus* L.) seeds, another important oil source in the world, were preferred as the materials for the present study. Seeds were disinfected by soaking in 10% NaClO solution for 10 min and then washed three times with distilled water (Yan et al. 2021). For both plants, 8 seeds were sown in each pot, and each treatment was performed in triplicate. Treatments included control, 3% sucrose, 75 mM NaCl, 150 mM NaCl, 75 mM NaCl + 3% sucrose, and 150 mM NaCl + 3% sucrose. Plants were grown in a growth chamber (DigiTech) installed at a photoperiod of 16/8 h, 24/20 °C day/night temperature, 4,200 lx light intensity, and 60% humidity. Plant samples were irrigated with distilled water for a month, after which it was as follows: the control group continued to be irrigated with distilled water every two days, while the remaining groups were irrigated with their respective treatments every two days. Sunflower and canola seedlings were harvested after 45 days of sowing. Growth parameters such as stem-root length (cm), stem-root fresh and dry weight (g), stem-root biomass (g m^-^^2^), and tolerance index were calculated.

### Determination of chlorophyll amounts

Chlorophyll *a*, *b*, and total chlorophyll amounts of 0.05 g fresh leaf samples, which were previously homogenized with 15 mL of 80% (v/v) acetone, were determined at 645 nm and 663 nm wavelengths according to the method of Arnon (1949).

### Determination of lipid peroxidation

Malondialdehyde (MDA) content, an indicator reflecting lipid peroxidation, was detected using the thiobarbituric acid (TBA) assay. The lipid peroxidation level of leaves was determined in terms of MDA content according to Sairam and Saxena (2000). In summary, 0.25 g of fresh leaf tissue was homogenized with 5 mL of 0.1% (w/v) trichloroacetic acid (TCA). The homogenate was centrifuged at 10,000 g for 5 min. To 500 μL of supernatant, 2 mL of 0.5% (w/v) TBA dissolved in 20% (w/v) TCA was added. The mixture was warmed at 95 °C for 30 min, cooled rapidly in an ice bath, and then centrifuged at 10,000 g for 10 min at 4 °C. MDA content was calculated by recording the supernatant absorbance at 532 nm and 600 nm.

### Determination of proline content

The proline content of the leaf samples was detected according to the method defined by Bates et al. (1973). In brief, 0.5 g of plant leaves from each treatment were homogenized in 10 mL of 3% (w/v) sulfosalicylic acid. The homogenate was then centrifuged at 10,000 g for 15 min; afterwards, 2 mL of the supernatant was incubated with 2 mL of acid ninhydrin and 2 mL of glacial acetic acid at 100 ºC for 1 h in a water bath and then cooled quickly in an ice bath. The colored reaction mixture was extracted with 4 mL of toluene, and the absorbance value was recorded at 520 nm using toluene as a blank. The proline concentration was determined using a calibration curve as μmol g^−1^ FW.

### Protein extraction and antioxidant enzyme assays

The plant leaves were weighed to 0.1 g and homogenized in 2 mL of extraction buffer consisting of 50 mM phosphate buffer, 1 mM EDTA, and 1% PVP. Then, this homogenate was centrifuged at 20,000 g for 20 min at 4 °C; the supernatant was used for the determination of antioxidant enzyme activity. In addition to the extraction buffer used in the determination of APX activity, 5 mM ascorbic acid was also included. Protein amounts of leaf samples were detected according to the method of Bradford (1976), using bovine serum albumin (BSA) as a standard. SOD enzyme activity was detected by spectrophotometrically measuring the inhibition of photoreduction of nitroblue tetrazolium (NBT) at 560 nm. One unit (U) of SOD activity is described as the amount of SOD enzyme that inhibits the photochemical reduction of NBT by 50% (Alici and Arabaci 2016). CAT activity was detected by spectrophotometric measurement of absorbance changes at a 240 nm wavelength as a result of the breakdown of hydrogen peroxide into water and oxygen by the action of the catalase enzyme, according to the method reported by Aebi (1984). APX activity was detected by measuring the decrease in the reaction rate at a 290 nm wavelength by following the method defined by Chaoui et al. (1997).

### Determination of gene expression of antioxidants

Total RNA isolation was performed from leaves of plant samples using two different kits. While the HibriGen Total RNA Isolation Kit was used for canola, the Macherey–Nagel Total DNA, RNA and Protein Isolation Kit was preferred for a more effective result in sunflower. Once the RNA samples were isolated, they were converted into cDNA using a cDNA synthesis kit [A.B.T.™ cDNA Synthesis Kit with RNase Inh. (High Capacity)]. For each gene, primers were designed to be used in quantitative real-time polymerase chain reaction (qRT-PCR). These primer sequences are provided in the supplementary material. The obtained cDNA samples were used as templates in quantitative real-time PCR. Before performing qRT-PCR experiments, the purity and quantity of cDNA samples were detected with a Nanodrop Spectrophotometer (AgileSpec) at 260 nm and 280 nm wavelengths, and some of the samples were diluted in the required amounts for qRT-PCR experiments. Quantitative real-time PCR was run in a PCR device (Agilent Technologies) using the instructions of the A.B.T.™ 2X qPCR SYBR Green Master Mix (ROX-free) kit. The conditions of the real-time PCR and all reagents were performed according to the kit instructions at 10 μl per reaction. The reaction cycle conditions included an initial denaturation step of 95 °C for 5 min, followed by 40 cycles of 95 °C for 30 s and 60 °C for 1 min. Following PCR amplification, the reactions were terminated with a program known as the melting curve. The melting curve program was 95 °C for 30 s, 65 °C for 30 s, followed by 30 s of increment from 65 °C to 95 °C.

### Statistical analysis

Each study of the respective treatment, including the control, was carried out in three independent replicates. The data obtained from all treatments were analyzed using two-way ANOVA with Tukey’s multiple comparisons test at the p < 0.05 level among mean values via the GraphPad program. Statistically significant values were indicated with different letters, and standard deviations (SD) were indicated with a “ ± ” sign.

## Results

### Changes in growth parameters

Forty-five days after sowing, samples of leaves and roots were harvested from sunflower and canola plants. Measurements taken included stem and root length (cm), fresh and dry weight of the stem and root (g), stem and root biomass (g m^-^^2^), as well as tolerance index. It was found that two salt concentrations (75 and 150 mM NaCl) caused chlorosis and subsequent drying in some of the leaves of both plants. In the groups where salt and sucrose were applied together, these negative effects were found to be less, and relatively ameliorative effects were observed compared to the groups where only salt was applied.

The results revealed that the growth parameters of the sunflower and canola plants were significantly different between the groups affected by salt stress and the control group (Tables 1 and 2). Salt treatments (75 and 150 mM NaCl) markedly reduced the phenological characteristics of both plants, such as stem-root length, stem-root fresh and dry weight, and stem-root biomass, compared to the control. The main ameliorative effect of sucrose in sunflower was significant for stem length, stem fresh and dry weights at 75 mM NaCl, but there was an increase, although it was not significant, at 150 mM NaCl. On the other side, sucrose significantly increased sunflower root length and root fresh weight at 150 mM NaCl, but interestingly decreased root fresh weight at 75 mM NaCl. However, the growth of canola seedlings limited by salinity stress was also effectively alleviated by sucrose application. This phenomenon was especially evidenced by an increasing trend in the fresh weights of the stem and root at 75 and 150 mM NaCl. Additionally, sucrose significantly increased canola stem dry weight and root length at 75 mM NaCl, while it increased canola root dry weight at 150 mM NaCl. For both plants, the effect of sucrose on biomass was that it increased stem biomass at both salt concentrations, but there was no change in root biomass. Exogenous application of sucrose mostly mitigated the negative effect of salt stress on the growth parameters of both plants. The salinity tolerance index (STI) calculated based on total plant length (stem + root) considerably decreased with increasing salinity concentration relative to the control (Table 3), while it was significantly increased in sucrose-treated sunflower and canola plants compared to the non-sucrose-treated plants under salinity. The salt tolerance index of sunflower was relatively higher than that of canola under all treatments.

**Table 1:** Phenological data of sunflower and canola stems

	Stem Length (cm)	Stem FW (g)	Stem DW (g)	Stem Biomass (g m^-^^2^)
*Sunflower*				
Control	54.5 ± 0.71a	18.15 ± 0.161b	2.860 ± 0.004a	89.4 ± 0.11b
Sucrose (Suc)	54.6 ± 0.39a	19.13 ± 0.336a	3.056 ± 0.056a	95.5 ± 1.73a
75 mM NaCl	36.0 ± 0.00c	8.802 ± 0.365e	1.606 ± 0.053c	50.2 ± 1.67e
150 mM NaCl	40.9 ± 1.98b	10.31 ± 0.127d	1.972 ± 0.003bc	61.6 ± 0.09d
75 mM NaCl + Suc	44.5 ± 0.71b	11.93 ± 0.147c	2.203 ± 0.011b	68.8 ± 0.33c
150 mM NaCl + Suc	42.9 ± 0.14b	10.77 ± 0.078d	2.226 ± 0.041b	69.6 ± 1.29c
*Canola*				
Control	30.8 ± 0.44a	7.911 ± 0.016a	1.071 ± 0.025a	33.5 ± 0.77a
Sucrose (Suc)	29.9 ± 0.21a	7.852 ± 0.007a	1.021 ± 0.006ab	31.9 ± 0.19b
75 mM NaCl	22.2 ± 0.24b	3.070 ± 0.066c	0.703 ± 0.010d	22.0 ± 0.31e
150 mM NaCl	21.8 ± 0.04b	3.007 ± 0.142c	0.786 ± 0.021cd	24.6 ± 0.66d
75 mM NaCl + Suc	22.5 ± 0.00b	4.004 ± 0.014b	0.899 ± 0.023bc	28.1 ± 0.71c
150 mM NaCl + Suc	21.7 ± 0.18b	4.163 ± 0.033b	0.925 ± 0.026abc	28.9 ± 0.82c

FW, Fresh Weight; DW, Dry Weight. Values are means ± SD of three independent replications. Different letters indicate significant differences (*p* < 0.05) among the treatments according to Tukey’s multiple comparisons test

**Table 2:** Phenological data of sunflower and canola roots

	Root Length (cm)	Root FW (g)	Root DW (g)	Root Biomass (g m^2 −1^)
*Sunflower*				
Control	40.3 ± 1.77a	3.511 ± 0.037b	0.435 ± 0.005a	13.6 ± 0.15ab
Sucrose (Suc)	40.0 ± 0.53a	3.753 ± 0.056a	0.439 ± 0.003a	13.7 ± 0.09a
75 mM NaCl	28.9 ± 0.57bc	2.908 ± 0.009c	0.350 ± 0.005ab	10.9 ± 0.16bc
150 mM NaCl	26.0 ± 1.41c	2.607 ± 0.023e	0.303 ± 0.007b	9.47 ± 0.20c
75 mM NaCl + Suc	25.5 ± 0.00c	2.132 ± 0.053f	0.326 ± 0.005b	10.2 ± 0.15c
150 mM NaCl + Suc	31.5 ± 2.12b	2.786 ± 0.037d	0.331 ± 0.001b	10.3 ± 0.02c
*Canola*				
Control	23.8 ± 0.97a	0.123 ± 0.001a	0.076 ± 0.001a	2.38 ± 0.09a
Sucrose (Suc)	24.0 ± 0.14a	0.125 ± 0.001a	0.080 ± 0.001a	2.50 ± 0.02a
75 mM NaCl	14.4 ± 0.76c	0.061 ± 0.003c	0.051 ± 0.002b	1.59 ± 0.15b
150 mM NaCl	15.6 ± 0.32bc	0.049 ± 0.003d	0.035 ± 0.001c	1.09 ± 0.02c
75 mM NaCl + Suc	16.6 ± 1.32b	0.069 ± 0.003c	0.048 ± 0.003b	1.50 ± 0.09bc
150 mM NaCl + Suc	16.7 ± 0.65b	0.092 ± 0.001b	0.045 ± 0.003b	1.41 ± 0.13bc

FW, Fresh Weight; DW, Dry Weight. Values are means ± SD of three independent replications. Different letters indicate significant differences (*p* < 0.05) among the treatments according to Tukey’s multiple comparisons test

**Table 3:** Chlorophyll *a*, *b*, total chlorophyll amounts and tolerance indices of the sunflower and canola leaves samples

	Chlorophyll *a* (mg/mL)	Chlorophyll *b* (mg/mL)	Total Chlorophyll (mg/mL)	Tolerance Index (%)
*Sunflower*				
Control	3.447 ± 0.039c	2.222 ± 0.089b	5.606 ± 0.195d	100a
Sucrose (Suc)	3.833 ± 0.063c	2.652 ± 0.411ab	6.572 ± 0.032c	99.84b
75 mM NaCl	1.777 ± 0.008d	1.735 ± 0.037c	3.495 ± 0.027e	68.50f
150 mM NaCl	1.480 ± 0.004d	1.443 ± 0.115c	2.922 ± 0.118f	70.61e
75 mM NaCl + Suc	5.254 ± 0.311a	2.716 ± 0.056a	7.925 ± 0.185a	73.88d
150 mM NaCl + Suc	4.498 ± 0.038b	2.585 ± 0.055ab	7.061 ± 0.100b	78.52c
*Canola*				
Control	5.917 ± 0.189b	5.612 ± 0.038a	11.564 ± 0.315a	100a
Sucrose (Suc)	6.429 ± 0.003a	4.614 ± 0.084b	11.040 ± 0.087b	98.63b
75 mM NaCl	3.125 ± 0.016de	4.680 ± 0.078b	7.803 ± 0.048d	66.85f
150 mM NaCl	2.909 ± 0.093e	3.451 ± 0.180c	6.358 ± 0.086e	68.41e
75 mM NaCl + Suc	3.387 ± 0.039d	4.595 ± 0.067b	7.979 ± 0.028d	71.52c
150 mM NaCl + Suc	4.652 ± 0.023c	4.274 ± 0.195b	8.923 ± 0.218c	70.15d

FW, Fresh Weight; DW, Dry Weight. Values are means ± SD of three independent replications. Different letters indicate significant differences (*p* < 0.05) among the treatments according to Tukey’s multiple comparisons test

### Changes in chlorophyll amounts

Both sunflower and canola plants grown under NaCl stress had significantly lower amounts of chlorophyll (Chl *a*, *b*, and total chlorophyll) relative to those in the control groups (Table 3). However, exogenously applied sucrose significantly increased the chlorophyll amounts in the salt-stressed sunflower seedlings relative to those of the salt-stressed seedlings without sucrose treatment. Even sucrose application with 75 and 150 mM of NaCl treatments increased the chlorophyll amounts more than the control in sunflower. The effect of sucrose in canola was a significant increase for all chlorophyll amounts at 150 mM NaCl, but there was no change at 75 mM NaCl.

### Changes in lipid peroxidation

**Fig. 1:**
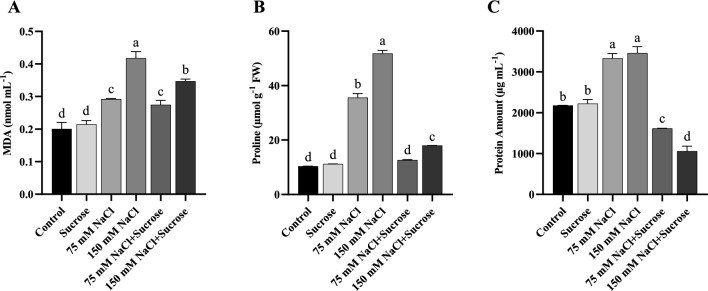
**A** MDA content, **B** Proline content and **C** Total protein amount of sunflower leaves. Data represent means of three independent replicates ± SD. Different letters in the data column indicate significant differences (*p* < 0.05) according to Tukey’s multiple comparisons test

**Fig. 2:**
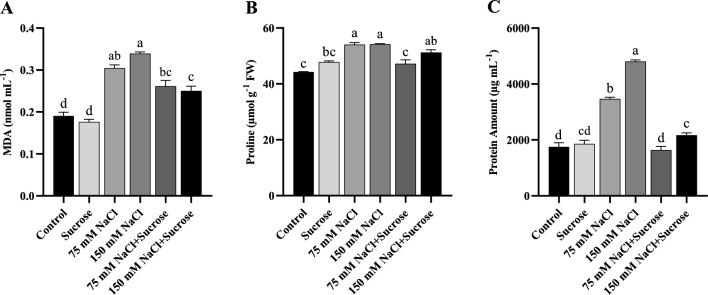
**A** MDA content, **B** Proline content and **C** Total protein amount of canola leaves. Data represent means of three independent replicates ± SD. Different letters in the data column indicate significant differences (*p* < 0.05) according to Tukey’s multiple comparisons test

Lipid peroxidation, representing oxidative damage, was detected as MDA content in the leaves of both sunflower and canola plants. MDA levels increased with the aggravation of salt stress, and the MDA content in the leaves of both salt-treated plants was much higher than that in the control group (Figs. 1A and 2A). Exogenous sucrose treatment at 150 mM NaCl concentration dramatically decreased MDA accumulation relative to only 150 mM NaCl-stressed sunflower and canola plants.

### Changes in proline contents

The proline content in sunflower and canola leaves was significantly affected by salt stress and sucrose application. In salt-treated sunflower plants, proline content was significantly increased relative to the control, with a higher level being attained at 150 mM NaCl (Fig. 1B). The proline content of canola plants increased similarly at both 75 and 150 mM NaCl concentrations (Fig. 2B). In contrast, proline content was considerably reduced by sucrose supplementation in sunflower, whereas there was not as sharp a decline in canola as in sunflower.

### Changes in total protein amount

The increase in the amount of protein in salt-treated sunflower and canola leaves was noted relative to the control (Figs. 1C and 2C). On the other side, the sucrose treatment at both salt concentrations significantly decreased the accumulation of protein, but more pronounced at 150 mM NaCl in both sunflower and canola plants.

### Changes in antioxidant enzyme activities

**Fig. 3:**
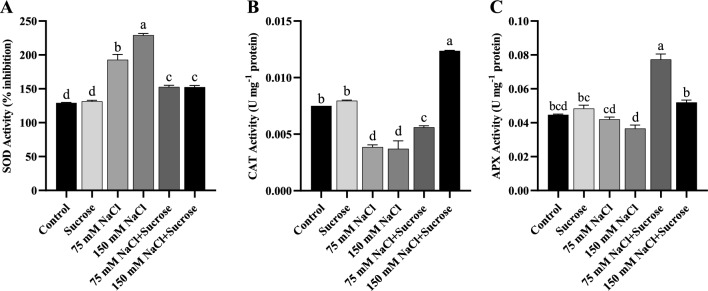
Antioxidant enzyme activities of sunflower leaf samples **A** SOD, **B** CAT, **C** APX. Data represent means of three independent replicates ± SD. Different letters in the data column indicate significant differences (*p* < 0.05) according to Tukey’s multiple comparisons test

**Fig. 4:**
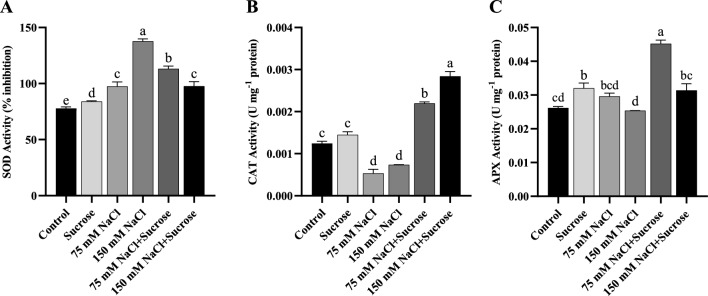
Antioxidant enzyme activities of canola leaf samples **A** SOD, **B** CAT, **C** APX. Data represent means of three independent replicates ± SD. Different letters in the data column indicate significant differences (*p* < 0.05) according to Tukey’s multiple comparisons test

To explore whether salt stress and sucrose treatment affected the antioxidant defense system in sunflower and canola, the activities of the main ROS-scavenging antioxidant enzymes, including SOD, CAT, and APX, were measured. For both sunflower and canola plants, compared to the control at both salt concentrations, SOD activity rised significantly (Figs. 3A and 4A) while CAT activity reduced markedly (Figs. 3B and 4B). Even though APX activity did not change statistically in both plants (Figs. 3C and 4C), it showed a reduction trend with increasing salt concentration in sunflower. In contrast, exogenously applied sucrose decreased the activity of SOD in the salt-treated sunflower plants relative to those of the stressed plants without sucrose (Fig. 3A). For canola, SOD activity increased at 75 mM NaCl with sucrose supplement and decreased at 150 mM NaCl with sucrose supplement, relative to only salt-applied samples (Fig. 4A). Although CAT and APX activities reduced or remained unchanged due to the salt stress, they increased considerably due to the exogenous treatment of sucrose in both plants (Figs. 3B-C and 4B-C). In short, exogenous sucrose combined with salt treatments enhanced the activity of CAT and APX enzymes but mostly decreased the SOD enzyme activity compared to that of the only salt-stressed plants, except for sucrose combined with 75 mM NaCl in canola.

### Changes in gene expression of antioxidants

**Fig. 5:**
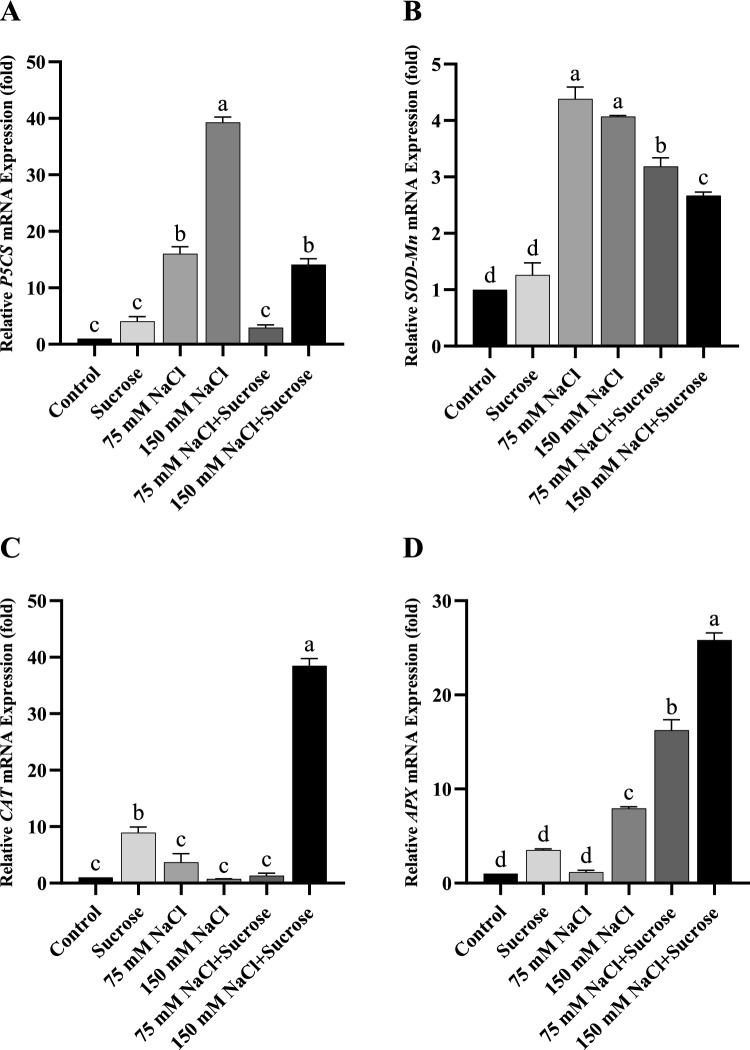
mRNA levels of sunflower leaf samples **A** *P5CS*, **B** *SOD-Mn*, **C** *CAT*, **D** *APX*. Data represent means of three independent replicates ± SD. Different letters in the data column indicate significant differences (*p* < 0.05) according to Tukey’s multiple comparisons test

In order to further investigate the effects of sucrose on the antioxidant defense system of sunflower and canola under salinity, the changes in the gene expression of antioxidants under different treatment conditions were examined. The expression of three antioxidant enzyme (*SOD*, *CAT*, *APX*) genes and pyrroline-5-carboxylate synthase (*P5CS*) gene in the leaves of sunflower and canola seedlings under salinity with sucrose treatment was analyzed by quantitative RT-PCR. According to the results obtained from sunflower, *P5CS* and *SOD-Mn* gene expressions increased significantly at both salt concentrations relative to the control (Fig. 5A and B). There was no significant change in *CAT g*ene expression in plants subjected to 75 and 150 mM NaCl relative to the control (Fig. 5C). In the same way, *APX* gene expression did not change at 75 mM NaCl concentration, but remarkably increased at 150 mM NaCl concentration compared to the control (Fig. 5D). On the other side, exogenously applied sucrose considerably reduced *P5CS* and *SOD* gene expression in salt-treated sunflower plants compared to those of the stressed plants without sucrose, whereas it significantly increased *APX* gene expression. Sucrose treatment did not significantly alter sunflower *CAT* gene expression at 75 mM NaCl, but dramatically increased it at 150 mM NaCl.

**Fig. 6:**
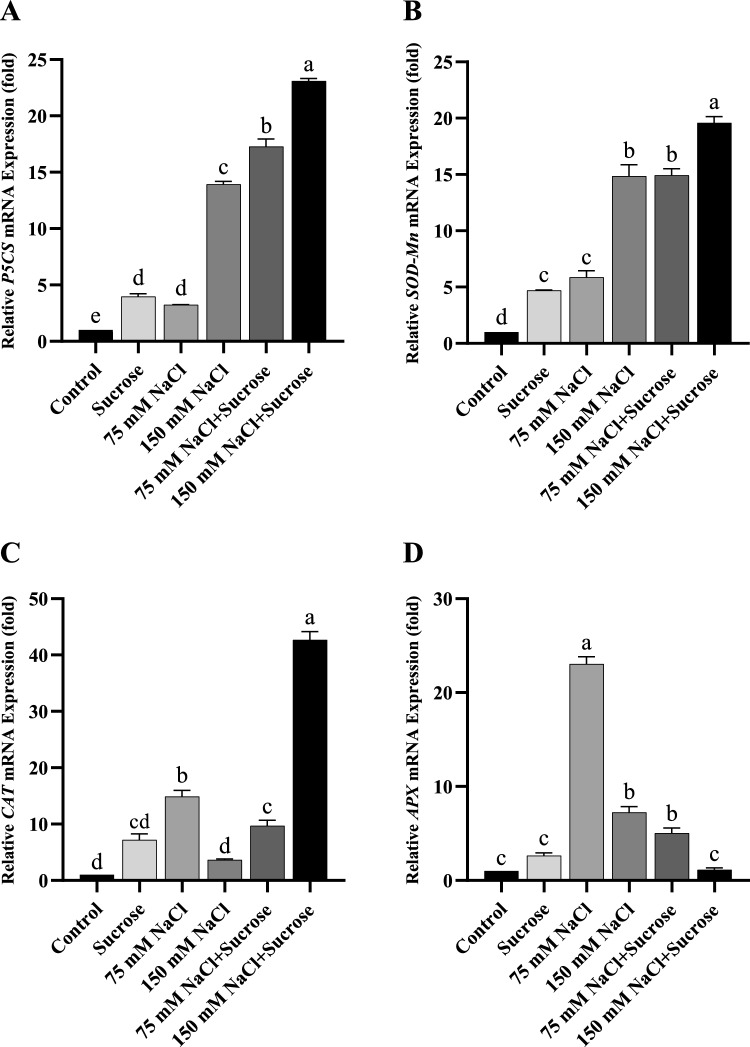
mRNA levels of canola leaf samples **A** *P5CS*, **B** *SOD-Mn*, **C** *CAT*, **D** *APX*. Data represent means of three independent replicates ± SD. Different letters in the data column indicate significant differences (*p* < 0.05) according to Tukey’s multiple comparisons test

In comparison with control, the expressions of *P5CS* and *SOD-Mn* in canola were significantly increased with the rise in salt levels (Fig. 6A and B). *APX* gene expression of canola also increased in both salt treatments (Fig. 6D), while its *CAT* gene expression increased only in 75 mM NaCl, but it was unchanged in 150 mM NaCl relative to control (Fig. 6C). On the other hand, treatment with sucrose led to much higher *P5CS* and *SOD* gene expression in salt-treated canola plants compared to those of the stressed plants without sucrose. Sucrose treatment decreased *APX* gene expression of canola at both salt levels, and also *CAT* gene expression at 75 mM NaCl. Interestingly, exogenous sucrose treatment conspicuously increased *CAT* gene expression of canola at 150 mM NaCl as in sunflower.

## Discussion

Limited plant growth and low productivity are most widespread effects in plants subjected to salt stress. Salinity can affect plant morphology and growth, resulting in yellowing and chlorosis of leaves, inhibition of root development, and reduced biomass (Muchate et al. 2016). The findings of the present study indicated that there were mild toxicity symptoms in the morphology of NaCl-treated sunflower and canola plants. Also, salt stress negatively affected the growth and biomass of sunflower and canola plants and resulted in a significant decline in their stem length, root length, stem-root fresh weights, and stem-root dry weights (Tables 1 and 2). Reductions in stem and root growth under salt conditions have been observed in several plants, including mint (Khorasaninejad et al. 2010), groundnut (Ambede et al. 2012), and alfalfa (Cornacchione and Suarez 2017). These reductions in stem and root lengths during salinity stress may be due to a decrease in cell division, cell elongation, and eventually cell growth. Moreover, the overaccumulation of sodium and chloride ions in the leaves through the transpiration stream may result in growth limitation via reduced leaf area, necrosis, yellowing, and shedding of leaves (Abdel-Farid et al. 2020).

Soluble sugars, such as sucrose, glucose, and trehalose, are mainly used as osmotic regulators to prevent dehydration and death of plant cells and to maintain the internal stability of the cells during salt exposure. Furthermore, sucrose, the primary product of photosynthesis, plays a fundamental role in carbon storage and is therefore associated with plant growth (Rosa et al. 2009). In this study, the application of sucrose markedly increased many growth parameters in sunflower and canola plants under salinity. Similar to our study, Wang et al. (2019) also found that the growth parameters of triticale seedlings treated with exogenous sugars, including glucose and sucrose, significantly increased. Salinity Tolerance Index (STI) is also an indicator reflecting the salt stress resistance of plants under saline conditions and gives an important idea about the ecological tolerance of these plants towards salinity. Consistent with our results, the tolerance index decreased with increasing salinity levels in some maize genotypes (Konuşkan et al. 2017). However, sucrose treatment enhanced the STI of sunflower and canola plants under salinity. This increase was higher in sunflower than in canola (Table 3).

The reduction in chlorophyll amounts of leaves is associated with salt stress-induced oxidative damage to chloroplasts (Ji et al. 2022). Low levels of chlorophyll under salt stress are a common phenomenon, and in this study, salt treatments significantly decreased the chlorophyll amounts in both sunflower and canola leaves (Table 3). Chlorophyll *a*, chlorophyll* b*, and total chlorophyll showed an overall declining trend with the increase of salt concentration. Broadly, the declining trend of chlorophyll amount under increasing NaCl concentration levels could be explained by the destruction of chlorophyll pigments, reduced chlorophyll synthesis, and instability of the pigment-protein complexes, as confirmed by similar findings by Rasool et al. (2013). On the other side, exogenous sucrose treatment significantly ameliorated the inhibition of chlorophyll *a*, chlorophyll* b*, and total chlorophyll amounts in the leaves of NaCl-treated sunflower and canola seedlings. Moreover, exogenous sucrose increased the chlorophyll amounts higher than the control in sunflower. Similarly, Siringam et al. (2012) stated that exogenous sucrose raised chlorophyll *a*, chlorophyll *b*, and total chlorophyll levels in rice exposed to salinity. The findings obtained from Noreen et al. (2019) also reported that several osmoprotectants enhanced chlorophyll levels in sunflower under salt stress, which were confirmed in this study.

MDA content, reflecting lipid peroxidation, is the commonly ascribed symptom of ROS-induced oxidative damage (Khan and Panda 2008). The findings of this study showed that two different NaCl treatments led to a notable increase in the levels of MDA in both sunflower and canola plants (Figs. 1A and 2A). Increased MDA contents in different plants under saline conditions were also demonstrated by Khoshgoftarmanesh et al. (2014), Taïbi et al. (2016), and Ji et al. (2022). Exogenous treatment of sucrose decreased the levels of MDA in salt-treated sunflower and canola plants; these reductions are significant at 150 mM NaCl treatments. Qiu et al. (2014) also stated that exogenous sucrose reduced MDA content in the *Arabidopsis* seedlings under salt stress, and our findings are in agreement with these results. Therefore, the sucrose treatment may be a beneficial way to protect plants from oxidative membrane damage caused by salinity.

To cope with the adverse effects of salinity-induced osmotic stress, plants accumulate high concentrations of compatible solutes, known as osmoprotectants, in their cytosol. Osmotic adjustment is an important mechanism that reduces cell water potential through increased net solute concentrations. This mechanism maintains cell turgor by decreasing the water potential of a cell without an accompanying decrease in cell turgor (Ashraf 2004). Therefore, it enables plants to tolerate salt stress in this way (Farhangi-Abriz and Torabian 2017). In this study, a similar accumulation trend of proline (Figs. 1B and 2B) and total proteins (Figs. 1C and 2C) was noted in sunflower and canola leaves under NaCl stress. Proline accumulates in large quantities under salt stress, as it is one of the most prevalent osmoprotectants in osmotic adjustment. In agreement with our study, Heidari (2012) and Fariduddin et al. (2013) found an enhancement in proline content of basil and cucumber plants under salt conditions, respectively. In contrast, sucrose, another important osmotic regulator, remarkably decreased proline content in salt-stressed sunflower but did not cause a sharp decline in salt-stressed canola plants as in sunflower. The reduction in proline content by exogenous sucrose can be explained by the osmoprotective effect of sucrose, which reduces the necessity to accumulate other osmolytes, such as proline, under salinity. The obvious increment in total protein levels in sunflower and canola under salinity has been suggested to indicate the synthesis of stress-responsive proteins. These proteins help detoxify ROS and thus play a role in stress adaptation (Ejaz et al. 2012). Increased accumulation of total proteins in response to saline stress has been reported by Abdul Qados (2011), Gerami et al. (2020), and Bano et al. (2021). On the other side, sucrose treatment caused a highly significant decrease in total protein amount of sunflower and canola plants under salinity. It is thought that exogenous sucrose may have exhibited osmoprotectant behavior like proline under salt stress, leading to a decline in the production of free amino acids such as proline and resulting in a decrease in total protein amount.

Plants are capable of dealing with the stressor by enhancing the synthesis of antioxidant metabolites, including proline. Besides that, antioxidant enzymes like SOD, CAT, and APX are also key enzymes for ROS scavenging in plants. The increases in the activity of these enzymes provide protection from oxidative damage; otherwise, the overaccumulation of ROS can cause cellular and molecular damage (Gill and Tuteja 2010; Saed-Moucheshi et al. 2014). Salinity may have different effects on the antioxidant enzymes of plants depending on factors such as the concentration and duration of salt stress, plant species, and variety. This situation has been addressed in many previous studies (Hernández and Almansa 2002; Al-Aghabary et al. 2005; Hediye Sekmen et al. 2007; Amirjani 2010; Nahar et al. 2015; Wang et al. 2017; Ramadan et al. 2019). As reported in similar findings obtained from Xu et al. (2013), the current study also demonstrated that under salinity, SOD activity remarkably increased in sunflower and canola plants, while CAT and APX activities did not increase (Figs. 3A-C and 4A-C). This indicated that the SOD enzyme plays a critical role in ROS detoxification in these plants under salinity stress. In plants, the SOD enzyme is the initial enzymatic defense against ROS and preserves cells by efficiently catalyzing the dismutation of the superoxide radical (O_2_^•−^) to hydrogen peroxide (H_2_O_2_). Hydrogen peroxide, the product of SOD activity, is highly detrimental to chloroplasts, nucleic acids, and proteins, and it must be eliminated by H_2_O_2_ detoxifying enzymes such as CAT and APX by reduction to water (Oueslati et al. 2010).

Sucrose treatment mostly reduced the activity of the SOD enzyme in salinity-treated seedlings, strongly suggesting that sucrose involved in the direct scavenging of O_2_^•−^ or in modulating SOD activity. However, since the activities of CAT and APX enzymes did not increase under salinity, hydrogen peroxide in sunflower and canola leaves may not be completely detoxified and may have accumulated, which was supported by the increase in MDA content. In particular, the decrease in CAT activity could lead to a severe inhibition of H_2_O_2_ detoxification in seedlings under salt stress. In contrast, sucrose treatment combined with salt stress provided a harmonious and balanced operation of the antioxidant enzymes by enhancing the activity of CAT and APX enzymes and regulating the SOD enzyme activity to the required level in sunflower and canola plants. The harmonious and balanced functioning of antioxidant enzymes is vitally important for plant survival during stress. It has been well documented in previous studies that antioxidant enzyme levels are altered under salt stress and that improved antioxidant capacity by sugars acting as osmoprotectants is directly associated with salt tolerance (Hu et al. 2012; Qiu et al. 2014; Yang et al. 2014; Mostofa et al. 2015). In this study, sucrose treatment effectively alleviated salinity-induced oxidative damage, as proven by the reduction of the lipid peroxidation product, MDA, in sunflower and canola leaves.

Plants have evolved an efficient antioxidant defense system in order to overcome the increasing levels of ROS during stress and maintain redox homeostasis. In this defense process, in addition to antioxidants, the regulation of genes encoding antioxidants at the mRNA level is a fundamental issue that cannot be ignored in ROS detoxification (Menezes-Benavente et al. 2004). In the literature, a limited number of studies have revealed that the gene expression of antioxidants is altered differently under salt stress (Kim et al. 2007; Rasool et al. 2013; Rossatto et al. 2017; Vighi et al. 2017). In this study, salt stress generally increased *P5CS*, *SOD-Mn,* and *APX* gene expressions in both plants, but did not cause a significant change in *CAT* gene expression except for the 75 mM NaCl treatment of canola (Figs. 5A-D and 6A-D). As a consequence, transcription levels of *P5CS*, a gene involved in proline synthesis, were strongly induced under salt stress, coinciding with a rise in proline content. Several studies have demonstrated that the expression of the *P5CS* gene is related to proline accumulation under salinity stress (Razavizadeh et al. 2009; Tavakoli et al. 2016). Likewise, for both plants, the elevated transcript levels of *SOD-Mn* at two salt concentrations coincided with an increase in SOD activities, although not in a similar pattern in sunflower. Sairam et al. (2005) also found that the *Mn-SOD*, a SOD isoform, contributed to the total SOD activity in wheat. On the other hand, exogenous sucrose treatment under saline conditions affected antioxidant gene expressions differently in sunflower and canola. In one of the limited number of previous studies, Yang et al. (2022) also stated that exogenously applied trehalose sugar altered transcript mRNA levels of antioxidants under salt stress. Sucrose treatment decreased *P5CS* and *SOD-Mn* gene expressions and increased *APX* gene expressions in the leaves of sunflower seedlings under salinity stress. Surprisingly, it increased *P5CS* and *SOD-Mn* gene expressions and decreased *APX* gene expressions in canola under salinity, unlike sunflower. The most interesting result obtained from this study was that sucrose treatment under 150 mM salt stress increased CAT activity and *CAT* gene expression in both plants in a highly concordant manner. In fact, this emphasizes the highly pronounced effect of sucrose on the CAT enzyme at high salinity in both plants.

## Conclusion

In this study, the role of sucrose in alleviating the impacts of salinity stress, which has become a serious ecological problem all over the world, was investigated in sunflower and canola, important oil crops. In summary, exogenously applying a low concentration of sucrose facilitated plant development and improved the stem biomass by increasing chlorophyll amounts in sunflower and canola seedlings under salt stress. Additionally, sucrose assumed an osmoprotective role by modulating levels of osmoregulatory substances such as proteins and proline. Moreover, exogenous sucrose improved the H_2_O_2_-scavenging capacity of sunflower and canola plants under salinity by enhancing the activities of antioxidant enzymes such as CAT and APX. Besides that, it also provided an effective and balanced antioxidant defense by regulating SOD activity. Taking all the results together, sucrose treatment supported improved growth performance and antioxidant defense by alleviating salt stress in sunflower and canola seedlings. This study showed that sucrose supplementation could be a potential tool to enhance the ability of plants to overcome the adverse effects of salt stress.

## Supplementary Information

Below is the link to the electronic supplementary material.

Supplementary file1 (DOCX 20 KB)

## Data Availability

All data generated or analyzed during this study are included in this article and its supplementary materials.

## References

[CR1] Abdel-Farid IB, Marghany MR, Rowezek MM, Sheded MG (2020) Effect of salinity stress on growth and metabolomic profiling of *Cucumis sativus *and *Solanum lycopersicum*. Plants 9:1626. 10.3390/plants9111626 33238519 10.3390/plants9111626 PMC7700630

[CR2] Abdul Qados AMS (2011) Effect of salt stress on plant growth and metabolism of bean plant *Vicia faba* (L.). J Saudi Soc Agric Sci 10:7–15. 10.1016/j.jssas.2010.06.002

[CR3] Aebi H (1984) [13] Catalase in vitro. In: Packer L (ed) Methods in Enzymology. Elsevier 10.1016/s0076-6879(84)05016-3 6727660

[CR4] Ahmad F, Singh A, Kamal A (2020) Osmoprotective Role of Sugar in Mitigating Abiotic Stress in Plants. In: Roychoudhury A, Tripathi DK (eds) Protective Chemical Agents in the Amelioration of Plant Abiotic Stress: Biochemical and Molecular Perspectives, 1st edn. Wiley

[CR5] Al-Aghabary K, Zhu Z, Shi Q (2005) Influence of silicon supply on chlorophyll content, chlorophyll fluorescence, and antioxidative enzyme activities in tomato plants under salt stress. J Plant Nutr 27:2101–2115. 10.1081/PLN-200034641

[CR6] Alici E, Arabaci G (2016) Determination of SOD, POD, PPO and CAT Enzyme Activities in *Rumex obtusifolius* L. ARRB 11:1–7. 10.9734/ARRB/2016/29809

[CR7] Ambede JG, Netondo GW, Mwai GN, Musyimi DM (2012) NaCl salinity affects germination, growth, physiology, and biochemistry of bambara groundnut. Braz J Plant Physiol 24:151–160. 10.1590/S1677-04202012000300002

[CR8] Amirjani MR (2010) Effect of salinity stress on growth, mineral composition, proline content, antioxidant enzymes of soybean. Am J Plant Physiol 5:350–360. 10.3923/ajpp.2010.350.360

[CR9] Arnon DI (1949) Copper enzymes in isolated chloroplasts Polyphenoloxidase in *Beta vulgaris*. Plant Physiol 24:1–15. 10.1104/pp.24.1.1 16654194 10.1104/pp.24.1.1 PMC437905

[CR10] Ashraf M (2004) Some important physiological selection criteria for salt tolerance in plants. Flora-Morphol, Distrib, Funct Ecol Plants 199:361–376. 10.1078/0367-2530-00165

[CR11] Bano S, Iqbal S, Naqvi B et al (2021) Antioxidant enzymes and germination pattern: upshot of high salinity on soluble protein and average weight of *Spinacia oleracea* (spinach) seedlings. AFSJ 20:112–122. 10.9734/afsj/2021/v20i330283

[CR12] Bates LS, Waldren RP, Teare ID (1973) Rapid determination of free proline for water-stress studies. Plant Soil 39:205–207. 10.1007/BF00018060

[CR13] Bradford MM (1976) A rapid and sensitive method for the quantitation of microgram quantities of protein utilizing the principle of protein-dye binding. Anal Biochem 72:248–254. 10.1016/0003-2697(76)90527-3 942051 10.1016/0003-2697(76)90527-3

[CR14] Chaoui A, Mazhoudi S, Ghorbal MH, El Ferjani E (1997) Cadmium and zinc induction of lipid peroxidation and effects on antioxidant enzyme activities in bean (*Phaseolus vulgaris* L.). Plant Sci 127:139–147. 10.1016/S0168-9452(97)00115-5

[CR15] Cornacchione MV, Suarez DL (2017) Evaluation of alfalfa (*Medicago sativa* l.) populations’ response to salinity stress. Crop Sci 57:137–150. 10.2135/cropsci2016.05.0371

[CR16] Ejaz B, Sajid ZA, Aftab F (2012) Effect of exogenous application of ascorbic acid on antioxidant enzyme activities, proline contents, and growth parameters of *Saccharum* spp. hybrid cv. HSF-240 under salt stress. Turk J Biol. 10.3906/biy-1201-37

[CR17] Farhangi-Abriz S, Torabian S (2017) Antioxidant enzyme and osmotic adjustment changes in bean seedlings as affected by biochar under salt stress. Ecotoxicol Environ Saf 137:64–70. 10.1016/j.ecoenv.2016.11.029 27915144 10.1016/j.ecoenv.2016.11.029

[CR18] Fariduddin Q, Khalil RRAE, Mir BA et al (2013) 24-Epibrassinolide regulates photosynthesis, antioxidant enzyme activities and proline content of *Cucumis sativus* under salt and/or copper stress. Environ Monit Assess 185:7845–7856. 10.1007/s10661-013-3139-x 23443638 10.1007/s10661-013-3139-x

[CR19] Gangola MP, Ramadoss BR (2018) Sugars Play a Critical Role in Abiotic Stress Tolerance in Plants. In: Wani SH (ed) Biochemical. Elsevier, Physiological and Molecular Avenues for Combating Abiotic Stress Tolerance in Plants

[CR20] Gerami M, Majidian P, Ghorbanpour A, Alipour Z (2020) *Stevia rebaudiana* Bertoni responses to salt stress and chitosan elicitor. Physiol Mol Biol Plants 26:965–974. 10.1007/s12298-020-00788-0 32377046 10.1007/s12298-020-00788-0 PMC7196603

[CR21] Gill SS, Tuteja N (2010) Reactive oxygen species and antioxidant machinery in abiotic stress tolerance in crop plants. Plant Physiol Biochem 48:909–930. 10.1016/j.plaphy.2010.08.016 20870416 10.1016/j.plaphy.2010.08.016

[CR22] Goyal A, Tanwar B, Sihag MK et al (2021) Rapeseed/Canola (*Brassica napus*) Seed. In: Tanwar B, Goyal A (eds) Oilseeds: Health Attributes and Food Applications. Springer Singapore, Singapore

[CR23] Hediye Sekmen A, Türkan İ, Takio S (2007) Differential responses of antioxidative enzymes and lipid peroxidation to salt stress in salt-tolerant *Plantago maritima* and salt-sensitive *Plantago media*. Physiol Plant 131:399–411. 10.1111/j.1399-3054.2007.00970.x 18251879 10.1111/j.1399-3054.2007.00970.x

[CR24] Heidari M (2012) Effects of salinity stress on growth, chlorophyll content and osmotic components of two basil (*Ocimum basilicum* L.) genotypes. Afr J Biotechnol 11:379–384. 10.5897/AJB11.2572

[CR25] Hernández JA (2019) Salinity tolerance in plants: trends and perspectives. IJMS 20:2408. 10.3390/ijms20102408 31096626 10.3390/ijms20102408 PMC6567217

[CR26] Hernández JA, Almansa MS (2002) Short-term effects of salt stress on antioxidant systems and leaf water relations of pea leaves. Physiol Plant 115:251–257. 10.1034/j.1399-3054.2002.1150211.x 12060243 10.1034/j.1399-3054.2002.1150211.x

[CR27] Hu M, Shi Z, Zhang Z et al (2012) Effects of exogenous glucose on seed germination and antioxidant capacity in wheat seedlings under salt stress. Plant Growth Regul 68:177–188. 10.1007/s10725-012-9705-3

[CR28] Isayenkov SV (2012) Physiological and molecular aspects of salt stress in plants. Cytol Genet 46:302–318. 10.3103/S0095452712050040

[CR29] Ji X, Tang J, Zhang J (2022) Effects of salt stress on the morphology, growth and physiological parameters of *Juglans microcarpa* L. Seedlings Plants 11:2381. 10.3390/plants11182381 36145780 10.3390/plants11182381 PMC9506368

[CR30] Khan MH, Panda SK (2008) Alterations in root lipid peroxidation and antioxidative responses in two rice cultivars under NaCl-salinity stress. Acta Physiol Plant 30:81–89. 10.1007/s11738-007-0093-7

[CR31] Khan N, Ali S, Zandi P et al (2020) Role of sugars, amino acids and organic acids in improving plant abiotic stress tolerance. PAKJBOT. 10.30848/PJB2020-2(24)

[CR32] Khorasaninejad S, Mousavi A, Soltanloo H et al (2010) The effect of salinity stress on growth parameters, essential oil yield and constituent of peppermint (*Mentha piperita* L.). World Appl Sci J 11:1403–1407

[CR33] Khoshgoftarmanesh AH, Khodarahmi S, Haghighi M (2014) Effect of silicon nutrition on lipid peroxidation and antioxidant response of cucumber plants exposed to salinity stress. Arch Agr Soil Sci 60:639–653. 10.1080/03650340.2013.822487

[CR34] Kim DW, Shibato J, Agrawal GK et al (2007) Gene transcription in the leaves of rice undergoing salt-induced morphological changes (*Oryza sativa* L.). Mol Cells 24:45–59. 10.1016/S1016-8478(23)10755-2 17846498

[CR35] Konuşkan Ö, Gözübenli H, Atiş İ, Atak M (2017) Effects of Salinity Stress on Emergence and Seedling Growth Parameters of Some Maize Genotypes (*Zea mays* L.). Turkish JAF SciTech. 5:1668–1672. 10.24925/turjaf.v5i12.1668-1672.1664

[CR36] Lunn JE (2016) Sucrose Metabolism. In: Hetherington AM (ed) Encyclopedia of Life Sciences, 1st edn. Wiley

[CR37] Menezes-Benavente L, Teixeira FK, Alvim Kamei CL, Margis-Pinheiro M (2004) Salt stress induces altered expression of genes encoding antioxidant enzymes in seedlings of a Brazilian indica rice (*Oryza sativa* L.). Plant Sci 166:323–331. 10.1016/j.plantsci.2003.10.001

[CR38] Mostofa MG, Hossain MA, Fujita M (2015) Trehalose pretreatment induces salt tolerance in rice (*Oryza sativa* L.) seedlings: oxidative damage and co-induction of antioxidant defense and glyoxalase systems. Protoplasma 252:461–475. 10.1007/s00709-014-0691-3 25164029 10.1007/s00709-014-0691-3

[CR39] Muchate NS, Nikalje GC, Rajurkar NS et al (2016) Plant salt stress: adaptive responses, tolerance mechanism and bioengineering for salt tolerance. Bot Rev 82:371–406. 10.1007/s12229-016-9173-y

[CR40] Munns R, Tester M (2008) Mechanisms of salinity tolerance. Annu Rev Plant Biol 59:651–681. 10.1146/annurev.arplant.59.032607.092911 18444910 10.1146/annurev.arplant.59.032607.092911

[CR41] Nahar K, Hasanuzzaman M, Alam MM, Fujita M (2015) Roles of exogenous glutathione in antioxidant defense system and methylglyoxal detoxification during salt stress in mung bean. Biologia Plant 59:745–756. 10.1007/s10535-015-0542-x

[CR42] Noreen S, Faiz S, Akhter MS, Shah KH (2019) Influence of Foliar Application of Osmoprotectants to Ameliorate Salt Stress in Sunflower (*Helianthus annuus* L). SJA. 10.17582/journal.sja/2019/35.4.1316.1325

[CR43] Oueslati S, Karray-Bouraoui N, Attia H et al (2010) Physiological and antioxidant responses of *Mentha pulegium* (Pennyroyal) to salt stress. Acta Physiol Plant 32:289–296. 10.1007/s11738-009-0406-0

[CR44] Parihar P, Singh S, Singh R et al (2015) Effect of salinity stress on plants and its tolerance strategies: a review. Environ Sci Pollut Res 22:4056–4075. 10.1007/s11356-014-3739-1 10.1007/s11356-014-3739-1 25398215

[CR45] Pirasteh-Anosheh H, Ranjbar G, Pakniyat H, Emam Y (2016) Physiological mechanisms of salt stress tolerance in plants: An overview. In: Azooz MM, Ahmad P (eds) Plant-Environment Interaction, 1st edn. Wiley

[CR46] Qiu ZB, Wang YF, Zhu AJ et al (2014) Exogenous sucrose can enhance tolerance of *Arabidopsis thaliana* seedlings to salt stress. Biologia Plant 58:611–617. 10.1007/s10535-014-0444-3

[CR47] Ramadan AA, Abd Elhamid EM, Sadak MSh (2019) Comparative study for the effect of arginine and sodium nitroprusside on sunflower plants grown under salinity stress conditions. Bull Natl Res Cent 43:118. 10.1186/s42269-019-0156-0

[CR48] Rasool S, Ahmad A, Siddiqi TO, Ahmad P (2013) Changes in growth, lipid peroxidation and some key antioxidant enzymes in chickpea genotypes under salt stress. Acta Physiol Plant 35:1039–1050. 10.1007/s11738-012-1142-4

[CR49] Rauf S, Jamil N, Tariq SA et al (2017) Progress in modification of sunflower oil to expand its industrial value. J Sci Food Agric 97:1997–2006. 10.1002/jsfa.8214 28093767 10.1002/jsfa.8214

[CR50] Razavizadeh R, Ehsanpour AA, Ahsan N, Komatsu S (2009) Proteome analysis of tobacco leaves under salt stress. Peptides 30:1651–1659. 10.1016/j.peptides.2009.06.023 19573571 10.1016/j.peptides.2009.06.023

[CR51] Rosa M, Prado C, Podazza G et al (2009) Soluble sugars: Metabolism, sensing and abiotic stress: A complex network in the life of plants. Plant Signal Behav 4:388–393. 10.4161/psb.4.5.8294 19816104 10.4161/psb.4.5.8294 PMC2676748

[CR52] Rossatto T, Do Amaral MN, Benitez LC et al (2017) Gene expression and activity of antioxidant enzymes in rice plants, cv. BRS AG, under saline stress. Physiol Mol Biol Plants 23:865–875. 10.1007/s12298-017-0467-2 29158635 10.1007/s12298-017-0467-2 PMC5671449

[CR53] Saed-Moucheshi A, Shekoofa A, Pessarakli M (2014) Reactive oxygen species (ROS) generation and detoxifying in plants. J Plant Nutr 37:1573–1585. 10.1080/01904167.2013.868483

[CR54] Sairam RK, Saxena DC (2000) Oxidative stress and antioxidants in wheat genotypes: possible mechanism of water stress tolerance. J Agron Crop Sci 184:55–61. 10.1046/j.1439-037x.2000.00358.x

[CR55] Sairam RK, Srivastava GC, Agarwal S, Meena RC (2005) Differences in antioxidant activity in response to salinity stress in tolerant and susceptible wheat genotypes. Biologia Plant 49:85–91. 10.1007/s10535-005-5091-2

[CR56] Sami F, Yusuf M, Faizan M et al (2016) Role of sugars under abiotic stress. Plant Physiol Biochem 109:54–61. 10.1016/j.plaphy.2016.09.005 27639065 10.1016/j.plaphy.2016.09.005

[CR57] Siringam K, Juntawong N, Cha-um S et al (2012) Salt tolerance enhancement in indica rice (“*Oryza sativa*” L. spp. indica) seedlings using exogenous sucrose supplementation. Plant Omics 5:52–59

[CR58] Taïbi K, Taïbi F, Ait Abderrahim L et al (2016) Effect of salt stress on growth, chlorophyll content, lipid peroxidation and antioxidant defence systems in *Phaseolus vulgaris* L. S Afr J Bot 105:306–312. 10.1016/j.sajb.2016.03.011

[CR59] Tavakoli M, Poustini K, Alizadeh H (2016) Proline accumulation and related genes in wheat leaves under salinity stress. JAST 18:707–716

[CR60] Vighi IL, Benitez LC, Amaral MN et al (2017) Functional characterization of the antioxidant enzymes in rice plants exposed to salinity stress. Biologia Plant 61:540–550. 10.1007/s10535-017-0727-6

[CR61] Wang Y, Gu W, Meng Y et al (2017) γ-Aminobutyric acid imparts partial protection from salt stress injury to maize seedlings by improving photosynthesis and upregulating osmoprotectants and antioxidants. Sci Rep 7:43609. 10.1038/srep43609 28272438 10.1038/srep43609 PMC5341084

[CR62] Wang LH, Li GL, Wei S et al (2019) Effects of exogenous glucose and sucrose on photosynthesis in triticale seedlings under salt stress. Photosynt. 57:286–294. 10.32615/ps.2019.030

[CR63] Wind J, Smeekens S, Hanson J (2010) Sucrose: Metabolite and signaling molecule. Phytochemistry 71:1610–1614. 10.1016/j.phytochem.2010.07.007 20696445 10.1016/j.phytochem.2010.07.007

[CR64] Xu R, Yamada M, Fujiyama H (2013) Lipid peroxidation and antioxidative enzymes of two turfgrass species under salinity stress. Pedosphere 23:213–222. 10.1016/S1002-0160(13)60009-0

[CR65] Yan F, Zhang J, Li W et al (2021) Exogenous melatonin alleviates salt stress by improving leaf photosynthesis in rice seedlings. Plant Physiol Biochem 163:367–375. 10.1016/j.plaphy.2021.03.058 33930628 10.1016/j.plaphy.2021.03.058

[CR66] Yang L, Zhao X, Zhu H et al (2014) Exogenous trehalose largely alleviates ionic unbalance, ROS burst, and PCD occurrence induced by high salinity in Arabidopsis seedlings. Front Plant Sci. 10.3389/fpls.2014.00570 25400644 10.3389/fpls.2014.00570 PMC4212613

[CR67] Yang Y, Yao Y, Li J et al (2022) Trehalose alleviated salt stress in tomato by regulating ROS metabolism, photosynthesis, osmolyte synthesis, and trehalose metabolic pathways. Front Plant Sci 13:772948. 10.3389/fpls.2022.772948 35360323 10.3389/fpls.2022.772948 PMC8963455

[CR68] Zulfiqar F, Akram NA, Ashraf M (2020) Osmoprotection in plants under abiotic stresses: new insights into a classical phenomenon. Planta 251:3. 10.1007/s00425-019-03293-1 10.1007/s00425-019-03293-1 31776765

