# Supplementary material for: Exogenous sucrose alleviates salt stress in sunflower (Helianthus annuus L.) and canola (Brassica napus L.) by modulating osmotic adjustment and antioxidant defense system
Source: Physiol Mol Biol Plants. 2025 Mar 19;31(3):405–18. doi: 10.1007/s12298-025-01571-9 (PMC12006602; doi:10.1007/s12298-025-01571-9)
Supplement: Supplementary file 1 — Supplementary file1 (DOCX 20 KB) [file 12298_2025_1571_MOESM1_ESM.docx]

**THE SUPPLEMENTARY MATERIAL**

**Supplementary Table.** Sequences of the primers used for qRT-PCR in sunflower and canola

| Gene Name | Primer | Sequence (5’ to 3’) |
| --- | --- | --- |
| Sunflower |  |  |
| *P5CS* | P5CS (F) | TGGCAATGGAACTGAAGGCA |
|  | P5CS (R) | TCCATCCCACCTCTTCCCAT |
| *SOD* | SOD-Mn (F) | CTGGAAAGAATCTTGCCCCTACTCGT |
|  | SOD-Mn (R) | CAACCAATTTTTCCATAGAACCAAAATG |
| *CAT* | CAT (F) | CTTCCCGCTTGAATGTGAAG |
|  | CAT (R) | CCGATTACATAAACCCATCATC |
| *APX* | APX (F) | TGGCGATGCCTATTGTAGAC |
|  | APX (R) | TCCTCGCAAAAATCGATAGC |
| *Actin* | Actin (F) | AGGGCGGTCTTTCCAAGTAT |
|  | Actin (R) | ACATACATGGCGGGAACATT |
| Canola |  |  |
| *P5CS* | P5CS (F) | GGTCCTCCAAGCGATCCTAA |
|  | P5CS (R) | GGAATGCCACCATAAGCTGC |
| *SOD* | SOD-Mn (F) | TCAATCCAGACCTTCACGCT |
|  | SOD-Mn (R) | TTGAACTTGATGGCGCTCTG |
| *CAT* | CAT (F) | AGAGGGTTGTTCATGCGAGA |
|  | CAT (R) | TGGTGTAAAACTTGACGGCG |
| *APX* | APX (F) | ACTAGTTAACTCTGGGGCCA |
|  | APX (R) | GCTTAGAACAACGTCACATAGC |
| *GAPDH* | GAPDH (F) | GCCGCTTCCTTCAACATCAT |
|  | GAPDH (R) | TCGCAGCTTTCTCGAGTCTA |
